# Development and validity of a mobile application prototype for hospital shift handover

**DOI:** 10.1590/0034-7167-2023-0173

**Published:** 2024-11-22

**Authors:** Luciana Pizolio Garcia Dematte, André Estevam Jaques, Carla Moretti de Souza, Martina Mesquita Tonon, Cátia Millene Dell’Agnolo, Cremilde Aparecida Trindade Radovanovic, Adriane Bochi Cândido, Milton Alejandro Jorquera Malebrán

**Affiliations:** I Universidade Estadual de Maringá. Maringá, Paraná, Brazil

**Keywords:** Health Technology, Software Validation, Hospitalization, Health Information Exchange, Mobile Applications, Tecnología de la Salud, Validación de Programas de Computación, Hospitalización, Intercambio de Información en Salud, Aplicaciones Móviles

## Abstract

**Objective:**

To develop and validate a mobile application prototype for nursing shift handover in a hospital inpatient unit.

**Methods:**

A methodological study of technological production, carried out from April 2020 to January 2022, for mobile application construction and validity through the Design Thinking methodology. The study involved the stages of prototype development and validity by experts.

**Results:**

The application for mobile nursing shift handover obtained a usability score of 79 points and a content validity coefficient of 0.7.

**Conclusions:**

The instrument obtained an excellent assessment according to usability and agreement among experts. However, future studies are needed to implement this technology in order to assess effectiveness, time optimization and failures during communication.

## RESUMEN

**Objetivo:**

Desarrollar y validar un prototipo de aplicación móvil para el cambio de turno de enfermería en la unidad de internación hospitalaria.

**Métodos:**

Estudio metodológico de producción tecnológica, realizado de abril de 2020 a enero de 2022, para la construcción y validación de una aplicación móvil mediante la metodología Design Thinking. El estudio abarcó las stages de desarrollo y validación del prototipo por parte de expertos.

**Resultados:**

La aplicación de cambio de turno de enfermería móvil obtuvo una puntuación de usabilidad de 79 puntos y un coeficiente de validez de contenido de 0,7.

**Conclusiones:**

El instrumento obtuvo una excelente evaluación según usabilidad y acuerdo entre expertos. Sin embargo, se necesitan futuros estudios para implementar esta tecnología con el fin de evaluar la efectividad, la optimización del tiempo y los fallos durante la comunicación.

## INTRODUCTION

Shift handover is considered an important communication channel among nurses, as it allows the exchange of information about direct or indirect patient care, in order to guarantee continuous and quality care^(1)^.

Communication failures during this time contribute to the incidence of injuries and adverse events (AEs), especially when information about patients’ health status is not shared completely and efficiently^(2-4)^.

Regarding written communication, a systematic review study carried out in Brazilian hospitals identified that the main failures involve the absence of professional identification, lack of information about the patient, illegible letters, spelling errors, incorrect terminology, non-standardized acronyms, erasures and use of concealers^(4)^. However, some strategies can be implemented to ensure effective communication during shift handover, such as standardization of information, in order to improve communication during transition of care^(5)^.

Among the various tools available for structured communication, the Situation, Background, Assessment, Recommendation (SBAR), through its mnemonic, guides shift handover in order to avoid communication errors, failures or forgetfulness^(6)^.

This tool, developed by a North American team from the Institute for Healthcare Improvement in 2007, consists of fulfilling four criteria. The first highlights what is currently happening with patients (Situation); the second describes what situations led to that moment (Background); the third mentions professionals’ opinion on the problem (Assessment); and the fourth refers to what could be done to correct identified problems (Recommendation)^(1)^.

Research carried out in a public hospital in the state of Minas Gerais to assess shift handover in a Pediatric Intensive Care Unit found that the use of this tool enabled guidance and agility in communication among professionals, prioritizing the most relevant information^(7)^.

Another strategy capable of improving health communication is the technology known as “mobile health” or “m-Health”. Strategies to help professionals, through the use of cell phones, tablets and personal digital assistants (PDAs), optimize time and organize information, avoiding failures and, consequently, reducing AEs, being compatible with iOS, Android and Windows platforms^(7,9)^. These devices perform the function of pocket computers, as they allow easy access to the internet and are relatively low cost^(10-12)^.

An integrative review study carried out in Brazil concluded that there are several applications capable of facilitating and optimizing nursing work^(13)^. However, although the use of these technologies in healthcare has contributed to effective communication, to date, there are no validity studies in the scientific literature of a mobile application (App) specific to nursing shift handover in the medical and surgical hospital sectors.

Thus, considering the relevance of shift handover in the care and management scope of hospital care and in search of improving communication among nurses in this moment of transition, through standardization of information conveyed, the present study is proposed. Hereby, we envisioned the development of an application prototype that assists professional nurses in shift handover in medical and surgical inpatient units.

## OBJECTIVE

To describe the development and validity process of an application prototype for shift handover in a hospital inpatient unit, in medical and surgical clinics.

## METHODS

### Ethical aspects

The research project was submitted to the *Universidade Estadual de Maringá* Research Ethics Committee and to the *Hospital Universitário Regional de Maringá* Academic Activities Regulation Commission (COREA - *Comissão de Regulamentação das Atividades Acadêmicas*), Paraná, and was approved. It is noteworthy that all ethical requirements were met, as determined by Resolution 466/2012 of the Brazilian National Health Council.

### Study design, period and place

This is a methodological study of technological production for mobile application prototype development and validity for shift handover in a hospital inpatient unit in medical and surgical clinics. The Design Thinking (DT) methodology, also known as double diamond, proposed by the British Design Council, was used, consisting of four stages (discover, define, develop and deliver)^(14)^.

The study was carried out from April 2020 to January 2022, in a public teaching hospital located in northwestern Paraná, in the medical and surgical clinical sectors. Two stages were covered: mobile application prototype development and validity.

### Stage 1 - Mobile application prototype development

Between April and May 2020, a search was carried out on the download platforms on the Google Play^®^ application websites (Google™ application store) and the Apple Store^®^ website (Apple™ application store) using the terms : “Shift handover”, “Nursing”, “Health communication”, “Hospitalization”, “Mobile application” and “Patient handoff”. The objective was to identify the existence of Apps used by nurses in inpatient units of medical and surgical clinics, but there was a lack of specific apps for shift handover in these sectors.

The next stage consisted of an integrative literature review, with the guiding question: “What are the studies related to the use of technological resources for shift handover in nursing in a hospital inpatient unit?”.

Subsequently, participant observation was carried out in the hospital, by monitoring the dynamics of shift handover among the nursing team, which enabled a better understanding of the information passed on during shift handover (morning, afternoon and night). It was observed that the information used during shift handover was recorded in a nursing book, however, there was no standard script to guide the handover.

In this stage, the items to be inserted for prototype development were also defined, together with the participation of nurses who worked in the medical clinic and surgical clinic sectors of the hospital. To construct and structure the checklist with relevant information that should be included in the application prototype, these professionals’ experiences in daily practice were taken into account.

For technology development, using a shift handover instrument developed by nurses, a service for graphic creation of an application prototype was contracted from a technology company.

### Stage 2 - Application prototype validity by experts

A total of 50 experts were invited, who were duly informed, via email, of the study objectives. After 11 of them were accepted, the link to download the application prototype and the assessment questionnaire were sent via Google Forms, using the e-health system usability scale: System Usability Scale (SUS).

### Sample, inclusion and exclusion criteria

In the first stage, development, the sample consisted of nurses working in the medical and surgical clinical sectors during the construction phase of an instrument using the SBAR tool. In the second phase, validity, only experts participated.

For the shift handover instrument construction using the SBAR tool phase, the sample was used for convenience. In this case, all participants had experience in hospital shift handover and worked in these sectors during the period in which the study was carried out, these being the inclusion criteria.

In the content validity phase, experts were selected by analyzing the *Curriculum Lattes*, on the “*Lattes* Platform”, choosing the option “*Buscar Currículo Lattes*” (Search *Curriculum Lattes*). In the “*Assunto*” (Subject) tab, “health information exchange”, “hospital care”, “health technology” was written. We chose to select the “Doctors” and “Other researchers” database according to Fehring criteria^(15)^.

Adapted Fehring criteria were followed. Therefore, experts with a doctoral degree in nursing (3 points), a doctoral degree in nursing with a thesis related to shift handover (5 points), a master’s degree in nursing (2 points), a master’s degree in nursing with a dissertation on shift handover (4 points), in addition of nursing specialists in shift handover (2 points) and experience as a nurse in shift handover (1 point per year), were included. Experts who, despite completing the 5 points of adapted Fehring criteria, did not respond or did not return the questionnaire within 30 days, were excluded.

### Study protocol

To prepare the content inserted in mobile application prototype, an instrument for nursing shift handover was developed in a checklist format. This instrument was structured with the help of nurses working in the hospitalization sectors of the medical and surgical clinic of the hospital where the study was carried out.

The SBAR methodology was chosen to create the prototype checklist, as it is recommended by the World Health Organization (WHO), through the Joint Commission International, as a method that enables effective communication during shift handover, promoting patient safety culture^(15,16)^. The checklist contained the most relevant information necessary to respond to the SBAR mnemonic, considering the situation patients are in, what medical diagnoses they received, place of hospitalization, history, history of previous illness, allergies, use of ongoing medications, assessments (clinical status, physical examination, with description of drains, probes, vital signs, use of oxygen, types of diet) and recommendations for the next shift (intercurrences, assessments and examinations completed and pending).

The assessment questionnaire using Google Forms^®^ was composed of three parts, the first being the Informed Consent Form (ICF). If experts agreed to participate, they would open the second part of the questionnaire, in which they would answer demographic and socioeconomic questions. The third part dealt with ten system usability questions.

Experts had the opportunity to download the prototype and handle it after reading basic instructions on how to use it. After their first contact with the application prototype, they answered ten questions to assess app usability, using SUS, developed by Brooke in the Digital Equipment Corporation laboratory^(17)^, in the United Kingdom. SUS contains ten applied questions to measure product usability and is the most used when it comes to assessing the coverage of attributes and quality criteria in usability of e-health products^(11)^.

### Analysis of results, and statistics

To assess prototype usability, SUS, developed by Brooke, was applied, which contains ten questions to measure product usability. Responses are scored from 1 to 5 (“strongly disagree”, “disagree”, “neutral”, “agree” and “strongly agree”) using the Likert scale, presenting aspects of agreement and difficulties in relation to the application prototype. The Likert scale (1932) makes it possible to identify acceptance meaning and intensity. Measurement in this format is the most accepted among researchers^(18)^.

For odd-numbered questions, the individual score is the grade received minus 1 and, for even-numbered questions, the grade received minus 5. All answers are added together and the result is multiplied by 2.5, obtaining the overall value. Application assessment according to score is divided as follows: 20.5 (worst imaginable); 21 to 31.5 (poor); 39 to 52.5 (median); 53 to 73.5 (good); 74 to 85.5 (excellent); and 86 to 100 (best imaginable)^(19)^.

Emphasizing the importance of validity by experts, the Content Validity Coefficient (CVC) was used based on the responses to the SUS questionnaire to assess agreement among experts who assessed the application prototype, quantifying and interpreting judgments^(20)^.

CVC calculation is carried out in five stages. In the first, based on experts’ scores (one to five), the mean (Me) score for each item is calculated. Then (second stage), based on Me, the initial CVC (iCVC) for each item is calculated, dividing by the maximum value that the question could receive for relevance or clarity. In the third stage, initial error probability (IEP) is calculated to discount possible biases of expert evaluators for each question. In this case, one (1) is divided by the number of expert evaluators, raised by the same number of evaluators. With this, the final CVC (fCVC) (fourth stage) of each item/question can be calculated by subtracting iCVC from IEP. The last stage is intended for the total CVC of the questionnaire (tCVC) for each of the characteristics (language clarity, practical relevance and theoretical relevance). It consists of subtracting the mean iCVC (MiCVC) from the mean IEP (MIEP). After applying the calculation, the literature considers questions that have between tCVC 0.7 and 0.8 to be acceptable^(19)^. Microsoft Excel^®^ version 2021 was used to analyze the collected data.

## RESULTS

The developed application prototype was designed with a main screen with login and password after registration was carried out. It was called “*Passando Plantão*”, according to experts’ suggestion (66.75%).

**Figure 1:**
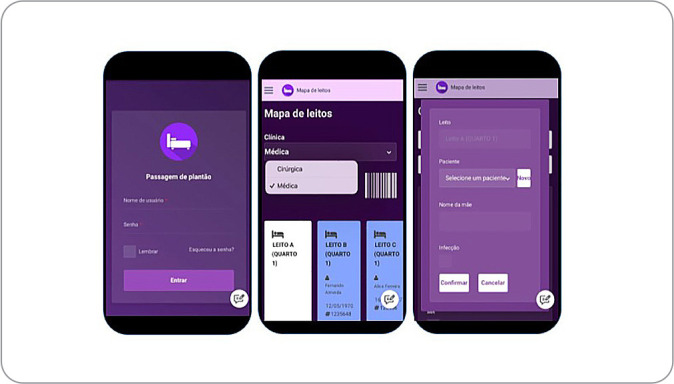
Home screen, bed overview screen and patient identification screen of the *Passando Plantão* application, Maringá, Paraná, Brazil, 2022

The screen allows an overview of beds available (white), occupied (blue) and with patients in isolation (orange). On the same bed map screen, the room and bed in which the patient is admitted, the mother’s name and medical record number are displayed, which will be filled in only once and will be saved for later use, as shown in Figure 1.

The application prototype enables shift handover systematization through the SBAR used to standardize information. Starting the SBAR mnemonic is patients’ Situation, with main and secondary medical diagnoses, doctor in charge and history of current illness (HCI). Subsequently, it is possible to view the Background, to fill in data on past history, use of medications and allergies. The next item, Assessment, mentions oxygen devices, food, edema, eliminations, probes, drains, infused drugs and vital signs. Lastly, there are the Recommendations, with space to record information about complications during the shift, requests for pending assessments, scheduled exams and/or scheduled surgeries with the date and type of procedure, in order to facilitate the necessary preparations.

**Figure 2:**
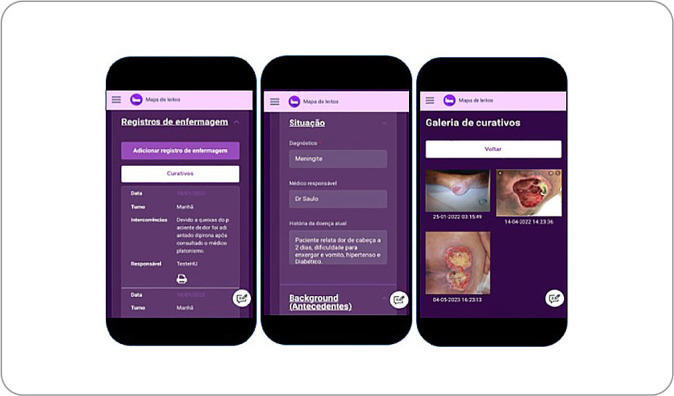
Nursing record screen, screen for entering patient information and dressing gallery screen of the *Passando Plantão* application, Maringá, Paraná, Brazil, 2022

In the Assessment item, it is also possible to include an image of the dressing, using the camera option, or attach images taken previously, for storage in a database. This allows to monitor patients’ progress, according to Figure 2.

All procedures that will be carried out can be registered on the home screen, where information will be stored so that, in the next registration, it will not be necessary to register it again. This is important information that includes, for instance, the use of a new medication, drains, diuresis, among others.

**Figure 3:**
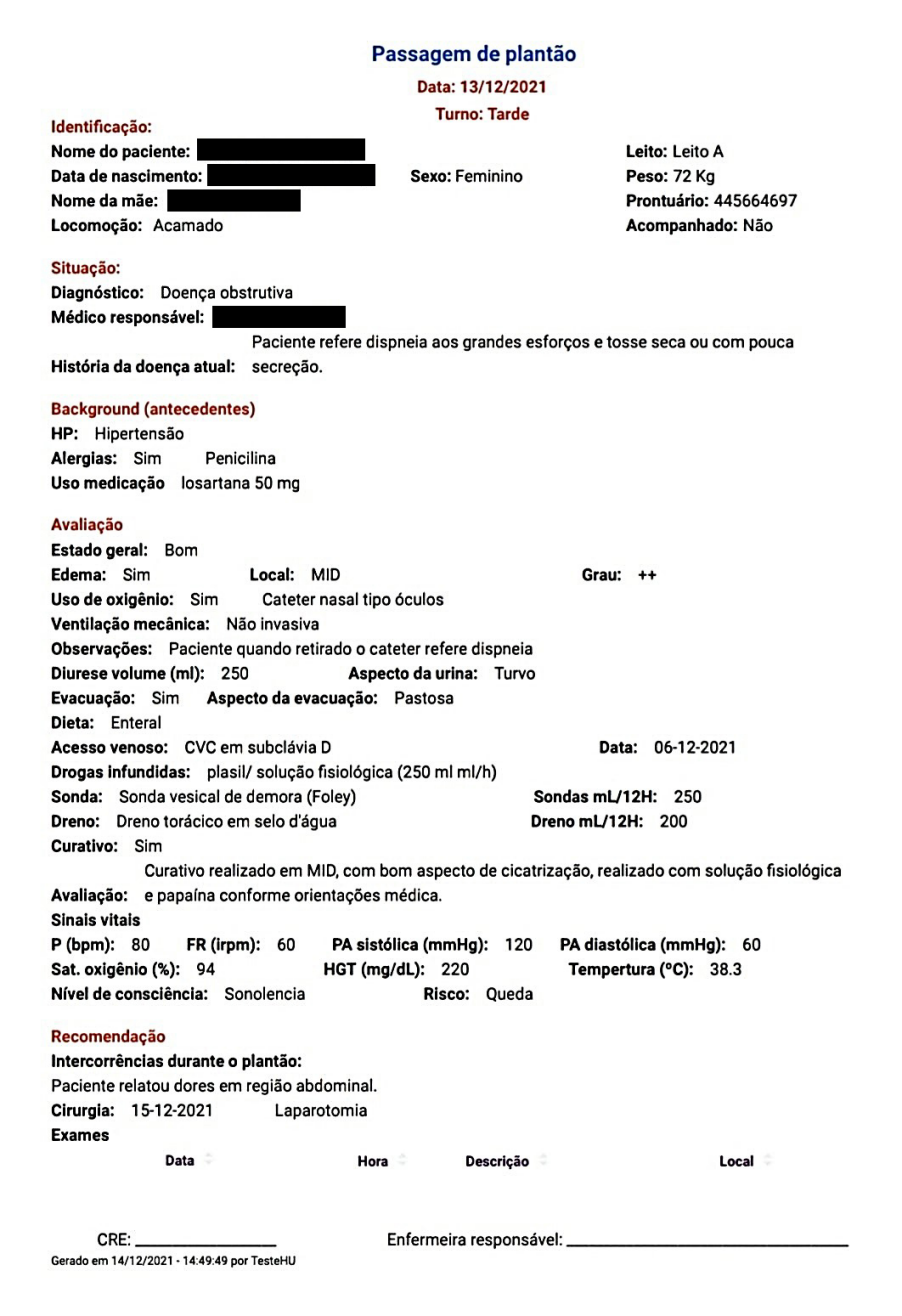
Nursing report screen in PDF format in the *Passando Plantão* application, Maringá, Paraná, Brazil, 2022

After completing the information, users can save the document or print it, generating a file in PDF format, as shown in Figure 3.

The application prototype content was validated by 11 experts. The majority were female (91.7%), married (75%), aged between 30 and 39 years (41.7%), 40 and 49 years (41.7%), from Paraná (83.3 %). The length of professional training ranged from 10 to 43 years, with an average of 18.2 years of study. A total of 75% of experts held a doctoral degree with experience and clinical practice in shift handover, and 41% worked in public hospitals (33.3%) and others (66.7%).

They assessed the App on the following aspects: complexity related to use, functionality, integrity of functions and difficulties, pointing out suggestions for improving content quality.

**Figure 4:**
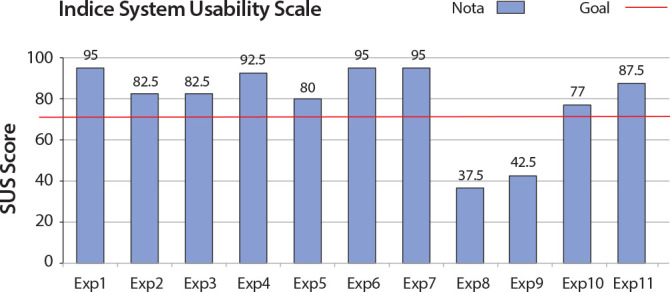
*Passando Plantão* prototype validity index as assessed by experts using the SUS scale. Maringá, Paraná, Brazil, 2022

The total mean SUS score was 79 points, considered an excellent classification for assessing product usability^(17)^, as shown in Figure 4.

The classification target used is a score of 68 points on the scale, with the values of this score being able to alternate according to experts’ answers. It was observed, among the 11 experts, that nine obtained answers above the average: 1, 2, 3, 4, 5, 6, 7, 10 and 11. Only two (experts 8 and 9) scored below the average, with 37.5 and 42.5, respectively.

Concerning the level of agreement of the answers, the CVC was used, as it assesses how many experts agreed or disagreed with the answers presented^(19)^. Question 4 obtained the lowest CVC index of 0.6. Considering the question “I think I would need technical assistance to use the application”, it is inferred that there may have been bias in the interpretation by the experts, as there was a lot of difference among the answers.

After analyzing all questions asked in Microsoft Office Excel, the total CVC of the questionnaire was 0.7, considered by the authors as acceptable for assessment, and should be between 0.7 and 0.8^(19)^, as shown in Chart 1.

**Chart 1:** Calculations of the SUS (System Usability Scale) questionnaire and Content Validity Coefficient

PARTICIPANTS	QUESTIONNAIRES									
	Q1	Q2	Q3	Q4	Q5	Q6	Q7	Q8	Q9	Q10
Expert 1	4	4	4	4	3	4	4	3	4	4
Expert 2	2	4	4	4	3	3	3	4	2	4
Expert 3	4	3	3	3	3	3	3	4	3	4
Expert 4	4	4	4	4	3	4	2	4	4	4
Expert 5	4	3	3	1	4	3	4	3	4	3
Expert 6	4	3	4	4	4	4	4	4	4	3
Expert 7	4	4	3	4	4	4	4	4	3	4
Expert 8	2	1	1	3	1	1	2	1	1	3
Expert 9	3	3	3	3	3	3	3	3	3	4
Expert 10	4	3	4	0	4	4	4	4	4	4
Expert 11	4	4	4	4	4	4	4	4	4	3
MEAN	3.5	3.3	3.4	3.1	3.3	3.4	3.4	3.5	3.3	3.6
iCVC	0.7	0.7	0.7	0.6	0.7	0.7	0.7	0.7	0.7	0.7
IEP	0	0	0	0	0	0	0	0	0	0
fCVC	0.7	0.7	0.7	0.6	0.7	0.7	0.7	0.7	0.7	0.7
Calculation of tCVC of the SUS questionnaire = 0.8 (iCVC mean -IEP mean)										

*Caption: iCVC: Initial Content Validity Coefficient; IEP: Initial Error Probability; fCVC: Final Content Validity Coefficient; tCVC: Total Content Validity Coefficient.*

## DISCUSSION

The prototype application developed was intended to facilitate nursing shift handover, through clear and organized information systematized by the SBAR method. The aim was to make communication effective, ensuring an effective communication process and, consequently, reducing AEs.

As for the use of the SBAR methodology for shift handover, two studies stand out^(21)^. The first, carried out to direct and speed up shift handover among nurse managers of a Pediatric Intensive Care Unit, enabled direction and agility in communication among nursing professionals, prioritizing the most important information. Furthermore, it allowed us to qualify and standardize the security items checked. The second, developed in two Intensive Care Units in Norway in 2016, on the use of the SBAR methodology in multidisciplinary communication, demonstrated that both teams noticed significant improvements in communication, safety and assertiveness in patient care.

With the increased use of Information and Communication Technology (ICTs) through the use of smartphones, tablets and computers, in various areas, including health, this tool has become a great ally in clinical decision-making and the quality of nursing care provision, among others^(22)^.

In this regard, the use of technology combined with service management improves communication among the team, optimizing time and organizing information on a full-time basis. The increase in technologies aimed at healthcare professionals is evident in recent years: it went from 82% in 2019 to 88% in 2021. This growth shows that, increasingly, professionals are looking to technology as a way to guarantee reliability, facilitate the flow of data and information, establish routines and protocols, in addition to qualifying the care process^(9)^.

The application prototype constitutes a technological innovation in health, as it becomes an App based on end users’ needs (target audience). The user-centered method establishes participation/collaboration among users and designers/researchers in the design phase for the development of computerized systems, recognizing them as participants in this construction process, which will impact adherence and future use of the application in their daily work lives^(21,22)^.

Nurses are receptive to acquiring new mobile technologies that help their work by optimizing time and reducing communication gaps. Mobile technologies facilitate systematization and access to information about patients and the care process^(23)^. In this regard, the prototype is evident and has this objective, with experts making its importance clear during shift handover.

*In principle, I believe that it meets what was proposed well. For shift handover, it is great.* (Expert 7)

*Self-explanatory application; it has all the information necessary for the nursing team shift handover.* (Expert 12)

In Brazil, several researchers have been working on the development of technologies that improve care and promote patient safety. Mention can be made of software development with the purpose of applying, in an easy and safe manner, the Nursing Process to patients assisted in an Intensive Care Unit, using as a basis the Theory of Basic Human Needs, by nurse Wanda de Aguiar Horta, adopting NANDA-International nursing diagnoses^(23)^.

Still in relation to the use of innovative technologies in the area of care with the aim of developing software for patient safety in pediatrics, a study concluded that the application of these tools favors the qualification of nursing care for pediatric patients, offering them more safety during hospitalization^(25)^.

Other applications in the literature demonstrate efficiency and good adherence to the use of these technologies. WebEnf used for nursing care is mentioned, which has 120 Standard Operating Procedures (SOPs) that address nursing care and procedures. This application was implemented in the public healthcare service of a large emergency in Rio de Janeiro and demonstrated feasibility and agility in finding protocols for preparing the material before starting the nursing procedure^(26)^.

Research assessed acceptance of a mobile application to prevent pressure injuries, early identifying patients at risk for pressure injuries through the application of the Waterlow scale and directing preventive measures according to the score. The use of the tool allowed professionals to optimize their time and plan the practice of actions in favor of effective and individualized prevention for each patient, promoting safe and quality care. Acceptance questionnaires revealed high application usability by professionals^(26)^.

Nurses, as managers, have the role of continuously assessing nursing services, looking for alternatives to improve the quality of care. It is known that the use of technology combined with service management improves communication among the team, optimizing time and organizing information on a full-time basis^(26)^.

Therefore, identifying technologies capable of improving communication among professionals in healthcare institutions has been essential. These technologies can only contribute to work processes, as they are seen as facilitators in the information communication and dissemination process.

### Study limitations

The difficulty of operationalization stands out, as it is a prototype, and its use may be difficult in sectors where the internet signal is not of good quality, especially for loading images. Another point to be considered was the fact that the instrument usability (in practice) was not assessed due to lack of time. However, this analysis should be carried out in a future study to examine time optimization, organization of information and nursing team assessment.

### Contributions to nursing

This study envisions benefits for professionals inserted in the job market, in order to facilitate the communication process among nurses, as it establishes systematized language during shift handover. The research developed also brings reflection on the importance of this digital resource usability in hospitals as a tool capable of boosting effective communication and decision-making.

## CONCLUSIONS

The mobile application prototype for nursing shift handover construction and validity contributes to better standardization and structuring of information with an emphasis on patients, which can improve the quality and safety of this information. The adoption of new technologies can help with communication among the team, favoring continuity of patient care. However, future studies are needed to implement the mobile application prototype in nurses’ practice, with the aim of assessing the effectiveness of information passed on, optimizing time and, consequently, reducing AEs caused by failures during communication.
