# Supplementary material for: Development and validity of a mobile application prototype for hospital shift handover
Source: Rev Bras Enferm. 2024 Nov 22;77(4):e20230173. doi: 10.1590/0034-7167-2023-0173 (PMC11654232; doi:10.1590/0034-7167-2023-0173)
Supplement: Translation

# Translation: Desenvolvimento e validação de protótipo de aplicativo móvel para passagem de plantão hospitalar

**DOI:** 10.1590/0034-7167-2023-0173pt

**Authors:** Luciana Pizolio Garcia Dematte, André Estevam Jaques, Carla Moretti de Souza, Martina Mesquita Tonon, Cátia Millene Dell’Agnolo, Cremilde Aparecida Trindade Radovanovic, Adriane Bochi Cândido, Milton Alejandro Jorquera Malebrán

**Affiliations:** I Universidade Estadual de Maringá. Maringá, Paraná, Brasil

**Keywords:** Tecnologia em Saúde, Validação de Software, Internação Hospitalar, Troca de Informação em Saúde, Aplicativos Móveis

## Abstract

**Objetivo:**

Desenvolver e validar um protótipo de aplicativo móvel para passagem de plantão de enfermagem na unidade de internação hospitalar.

**Métodos:**

Estudo metodológico de produção tecnológica, realizado no período de abril de 2020 a janeiro de 2022, para construção e validação de aplicativo móvel através da metodologia de *Design Thinking*. O estudo envolveu as etapas de desenvolvimento e validação do protótipo por especialistas.

**Resultados:**

O aplicativo móvel para passagem de plantão de enfermagem obteve pontuação de 79 pontos de usabilidade e coeficiente de validade de conteúdo de 0,7.

**Conclusões:**

O instrumento obteve excelente avaliação de acordo com a usabilidade e concordância entre os especialistas. No entanto, são necessários estudos futuros para implementação desta tecnologia, a fim de avaliar a eficácia, otimização do tempo e falhas durante a comunicação.

## INTRODUÇÃO

A passagem de plantão é considerada um importante canal de comunicação entre os enfermeiros, pois permite a troca de informações sobre a assistência direta ou indireta ao paciente, a fim de garantir uma assistência contínua e de qualidade^(1)^.

Falhas de comunicação durante esse momento contribuem para a incidência de agravos e eventos adversos (EAs), especialmente quando as informações sobre o estado de saúde do paciente não são compartilhadas de maneira completa e eficiente^(2-4)^.

Com relação à comunicação escrita, estudo de revisão sistemática realizado em hospitais brasileiros identificou que as principais falhas envolvem ausência de identificação do profissional, escassez de informações sobre o paciente, letras ilegíveis, erros de ortografia, terminologia incorreta, siglas não padronizadas, rasuras e uso de corretivos^(4)^. Contudo, algumas estratégias podem ser implementadas para garantir uma comunicação efetiva durante a passagem de plantão, tais como a padronização das informações, no sentido de melhorar a comunicação durante a transição do cuidado^(5)^.

Dentre as várias ferramentas disponíveis para comunicação estruturada, a *Situation, Background, Assessment, Recommendation* (SBAR), por meio do seu mnemônico, norteia a passagem de plantão de forma a evitar erros de comunicação, falhas ou esquecimentos^(6)^.

Tal ferramenta, desenvolvida por uma equipe norte-americana do *Institute for Healthcare Improvement* no ano de 2007, consiste no preenchimento de quatro critérios. O primeiro evidencia o que está acontecendo no momento atual com o paciente (*Situation*); o segundo descreve quais situações levaram àquele momento (*Background*); o terceiro menciona a opinião do profissional sobre o problema (*Assessment*); e o quarto remete ao que poderia ser feito para corrigir os problemas identificados (*Recommendation*)^(1)^.

Pesquisa realizada em um hospital público do estado de Minas Gerais para avaliar a passagem de plantão em uma unidade de terapia intensiva pediátrica constatou que o uso desta ferramenta possibilitou direcionamento e agilidade na comunicação entre os profissionais, priorizando as informações mais relevantes^(7)^.

Outra estratégia capaz de melhorar a comunicação em saúde é a tecnologia conhecida como “*mobile health*” ou “*m-Health*”. Estratégias para auxiliar os profissionais, através do uso do celular, *tablet* e assistentes pessoais digitais (PDAs), otimizam tempo e organizam informações, evitando falhas e, consequentemente, redução dos EAs, sendo compatíveis com as plataformas *IOS, Android e Windows* ^(7,9)^. Esses aparelhos desempenham a função de computadores de bolso, pois permitem acesso fácil à internet e possuem custo relativamente baixo^(9-11)^.

Estudo de revisão integrativa realizado no Brasil concluiu que existem vários aplicativos capazes de facilitar e otimizar o trabalho de enfermagem^(11,12)^. No entanto, embora o uso dessas tecnologias na saúde tenha contribuído para eficácia na comunicação, não há na literatura científica, até o presente momento, estudos de validação de aplicativo móvel (App) específico para passagem de plantão de enfermagem nos setores hospitalares médico e cirúrgico.

Assim, considerando a relevância da passagem de plantão no âmbito assistencial e gerencial da atenção hospitalar e em busca de melhorar a comunicação entre os enfermeiros nesse momento de transição, mediante a padronização das informações transmitidas, propõe-se o presente estudo. Vislumbra-se, por meio deste, o desenvolvimento de um protótipo de aplicativo que auxilie o profissional enfermeiro na passagem de plantão em unidades de internação médicas e cirúrgicas.

## OBJETIVO

Descrever o processo de desenvolvimento e validação de um protótipo de aplicativo para passagem de plantão em unidade de internação hospitalar, nas clínicas médica e cirúrgica.

## MÉTODOS

### Aspectos éticos

O projeto de pesquisa foi submetido ao Comitê de Ética em Pesquisa envolvendo Seres Humanos da Universidade Estadual de Maringá e à Comissão de Regulamentação das Atividades Acadêmicas (COREA) do Hospital Universitário Regional de Maringá Paraná, sendo aprovado. Destaca-se que todas as exigências éticas foram cumpridas, conforme determina a Resolução nº 466/2012 do Conselho Nacional de Saúde.

### Desenho, período e local do estudo

Estudo metodológico de produção tecnológica, para desenvolvimento e validação de um protótipo de aplicativo móvel para passagem de plantão em unidade de internação hospitalar, das clínicas médica e cirúrgica. Utilizou-se a metodologia de *Design Thinkings* (DT), também conhecida como diamante duplo, proposta pela *British Design Council*, sendo composta por quatro etapas (descobrir, definir, desenvolver e entregar)^(11)^.

O estudo foi realizado no período de abril de 2020 a janeiro de 2022, em um hospital de ensino de caráter público situado no Noroeste do Paraná, nos setores de clínica médica e cirúrgica. Foram percorridas duas etapas: desenvolvimento e validação do protótipo de aplicativo móvel.

### Etapa 1 - Desenvolvimento do protótipo de aplicativo móvel

Entre os meses de abril e maio de 2020, realizou-se uma pesquisa nas plataformas de *downloads* nos sites de aplicativos do Google Play® (Loja de aplicativos da Google™) e no site da Apple Store® (Loja de aplicativos da Apple™) utilizando os termos: “Passagem de Plantão”, “Enfermagem”, “Comunicação em saúde”, “Hospitalização”, “Aplicativo móvel” e “Transferência de paciente”. O objetivo foi identificar a existência de App utilizados por enfermeiros de unidades de internação de clínicas médicas e cirúrgicas, mas verificou-se a inexistência de aplicativos específicos para a passagem de plantão nesses setores.

A próxima etapa consistiu na revisão integrativa da literatura, tendo como pergunta norteadora: “Quais as publicações relacionadas ao uso de recursos tecnológicos para passagem de plantão em enfermagem em unidade de internação hospitalar?”.

Posteriormente, realizou-se a observação participante no hospital, por meio do acompanhamento da dinâmica da passagem de plantão entre a equipe de enfermagem, o que possibilitou melhor compreensão das informações repassadas durante a troca de turnos, matutino, vespertino e noturno. Observou-se que as informações utilizadas durante a passagem de plantão eram registradas no livro de enfermagem, não havendo, contudo, um roteiro-padrão para nortear o repasse destas.

Nessa etapa, também foram definidos os itens a serem inseridos para o desenvolvimento do protótipo, juntamente com a participação de enfermeiros que atuavam nos setores de clínica médica e clínica cirúrgica do hospital. Para construção e estruturação do *checklist* com informações relevantes que deveriam constar do protótipo de aplicativo, foram levadas em consideração as experiências desses profissionais na prática diária.

Para o desenvolvimento da tecnologia, utilizando um instrumento de passagem de plantão elaborado pelos enfermeiros, contratou-se o serviço de criação gráfica do protótipo de aplicativo de uma empresa de tecnologia.

### Etapa 2 - Validação do protótipo de aplicativo por especialistas.

Foram convidados 50 especialistas, os quais foram devidamente informados, via correio eletrônico, dos objetivos do estudo. Após o aceite de 11 deles, enviou-se o *link* para *download* do protótipo do aplicativo e o questionário de avaliação pelo *Google Forms*, utilizando a escala de usabilidade de sistema *e-health*, a *System Usability Scale* (SUS).

### Amostra, critérios de inclusão e exclusão

Na primeira etapa, desenvolvimento, a amostra foi constituída por enfermeiros atuantes nos setores de clínica médica e cirúrgica durante a fase de construção de um instrumento utilizando a ferramenta SBAR. Da segunda fase, de validação, participaram somente especialistas.

Para a fase de construção do instrumento de passagem de plantão utilizando a ferramenta SBAR, a amostra foi por conveniência. Neste caso, todos os participantes tinham experiência em passagem de plantão hospitalar e atuavam nesses setores durante o período em que o estudo foi realizado, sendo estes os critérios de inclusão.

Na fase de validação do conteúdo, os especialistas foram selecionados mediante análise do currículo Lattes, na “Plataforma Lattes” escolhendo-se a opção “Buscar Currículo Lattes”. No quadro “Assunto”, foi escrito: “troca de informação em saúde”, “cuidados hospitalares”, “tecnologia em saúde”. Optou-se por selecionar a base “Doutores” e “Demais pesquisadores”, de acordo com os critérios de *Fehring* ^(15)^.

Seguiram-se os critérios de *Fehring*, adaptados: Doutorado em enfermagem (3 pontos), Doutorado em enfermagem com tese relacionada à passagem de plantão (5 pontos); Mestre em enfermagem (2 pontos), Mestre em enfermagem com dissertação a respeito da passagem de plantão (4 pontos); Especialistas em enfermagem na área de passagem de plantão (2 pontos); e Experiência como enfermeiro na área de passagem de plantão (1 ponto por ano). Constituíram critérios de exclusão: especialistas que, mesmo completando os 5 pontos dos critérios de *Fehring* adaptados, não respondessem ou não devolvessem o questionário no prazo de 30 dias.

### Protocolo do estudo

Para elaboração do conteúdo inserido no protótipo de aplicativo móvel, desenvolveu-se um instrumento para passagem de plantão de enfermagem em formato de *checklist.* Tal instrumento foi estruturado com auxílio dos enfermeiros atuantes nos setores de internação de clínica médica e cirúrgica do hospital onde o estudo foi realizado.

Optou-se pela metodologia SBAR para a criação do *checklist* do protótipo, por ser recomendada pela Organização Mundial de Saúde (OMS) através da *Joint Commission Internacional*, como um método que possibilita a comunicação eficaz durante as trocas de turno, fomentando a cultura de segurança ao paciente^(16)^. O *checklist* continha as informações mais relevantes necessárias para responder ao mnemônico SBAR, considerando a situação em que o paciente se encontra, quais diagnósticos médicos recebeu, local de internação, antecedentes, história de doença pregressa, alergias, uso de medicamentos contínuos, avaliações (estado clínico, exame físico, com descrição de drenos, sondas, sinais vitais, uso de oxigênio, tipos de dieta) e recomendações para o próximo plantão (intercorrências, avaliações e exames realizados e pendentes).

O questionário de avaliação pelo *Google Forms®* foi composto por três partes, sendo a primeira o Termo de Consentimento Livre Esclarecido (TCLE). Caso o especialista aceitasse participar, abriria a segunda parte do questionário, na qual responderia a questões demográficas e socioeconômicas. A terceira parte versava sobre 10 questões de usabilidade do sistema.

Os especialistas tiveram a oportunidade de baixar o protótipo e manuseá-lo, após leitura de orientações básicas de como usá-lo. Depois do primeiro contato com o protótipo de aplicativo, eles responderam a 10 questões para avaliação da usabilidade do App, com o instrumento *System Usability Scale* (SUS), desenvolvido por Brooke, no laboratório da *Digital Equipment Corporation* ^(17)^, no Reino Unido. O SUS contém 10 questões aplicadas para medir a usabilidade do produto e é o mais empregado em se tratando de avaliação de cobertura de atributos e critérios de qualidade em usabilidade dos produtos *e-health* ^(11)^.

### Análise dos resultados e estatística

Para avaliar a usabilidade do protótipo, aplicou-se o questionário *System Usability Scale* (SUS) desenvolvido por Brooke, o qual contêm dez questões para medir a usabilidade de um produto. As respostas recebem pontuação de 1 a 5 (“discordo plenamente”; “discordo”, “neutro”; “concordo” e “concordo plenamente”) utilizando a escala de Likert, apresentando os aspectos de concordância e dificuldades em relação ao protótipo de aplicativo. A escala de Likert (1932) possibilita identificar o sentido e a intensidade da aceitação. A mensuração neste formato é a mais aceita entre os pesquisadores^(18)^.

Para as questões ímpares, o escore individual é a nota recebida menos 1 e, para as pares, a nota recebida menos 5. Somam-se todas as respostas e multiplica-se o resultado por 2,5, obtendo-se o valor global. A avaliação do aplicativo divide-se da seguinte forma: escores de 20,5 (pior imaginável); 21 a 31,5 (pobre); 39 a 52,5 (mediano); 53 a 73,5 (bom); 74 a 85,5 (excelente); e 86 a 100 (melhor imaginável) ^(19)^.

Enfatizando a importância da validação por especialistas, utilizou-se o coeficiente de validade de conteúdo (CVC) com base nas respostas do questionário SUS para avaliar a concordância entre os especialistas que avaliaram o protótipo de aplicativo, quantificando e interpretando os julgamentos^(19)^.

O cálculo do CVC é realizado em cinco etapas. Na primeira, com base nas notas dos especialistas (um a cinco), calcula-se a média das notas de cada item (Me). Em seguida (segunda etapa), com base na média (Me), calcula-se o CVC inicial para cada item (CVCi), dividindo-se pelo valor máximo que a questão poderia receber de pertinência ou clareza. Na terceira etapa, calcula-se a probabilidade de erro inicial (Pei), para descontar possíveis vieses dos especialistas avaliadores, para cada questão. Neste caso, divide-se um (1) pelo número de especialistas avaliadores, elevado pelo mesmo número de avaliadores. Com isso, o CVC final (quarta etapa) de cada item/questão (CVCc) poderá ser calculado a partir da subtração do CVCi pelo Pei. A última etapa é destinada para o CVC total do questionário (CVCt), para cada uma das características (clareza de linguagem, pertinência prática e relevância teórica). Consiste em subtrair a média do CVCi (MCVCi) pela média do Pei (MPei). Após a aplicação do cálculo, a literatura considera aceitáveis as questões que tiverem entre CVCt 0,7 e 0,8^(20)^. Utilizou-se, para análise dos dados coletados, o programa *Excel® (Microsoft)* versão 2021.

## RESULTADOS

O protótipo de aplicativo desenvolvido foi idealizado com uma tela principal com *login* e senha após o cadastro ser realizado. Foi denominado “Passando plantão”, de acordo com a sugestão dos especialistas (66,75%).

A tela permite uma visão geral sobre os leitos disponíveis (branco), ocupados (azul) e com pacientes em isolamento (laranja). Na mesma tela de mapa de leito estão apresentados o quarto e o leito em que o paciente está internado, nome da mãe e número do prontuário, que será preenchido somente uma vez e ficará salvo para uso posterior, conforme Figura 1.

**Figura 1:** Tela inicial, tela visão geral dos leitos e tela de identificação do paciente do aplicativo Passando Plantão, Maringá, Paraná, Brasil, 2022

O protótipo de aplicativo possibilita a sistematização da passagem de plantão através do mnemônico *SBAR* (Situação, Antecedentes, Avaliação e Recomendação de enfermagem) utilizado para padronizar as informações. Dando início ao mnemônico SBAR, está (Situação) do paciente, com diagnósticos médicos principal e secundários, médico responsável e história da doença atual (HDA). Na sequência, é possível visualizar os Antecedentes, para preenchimento de dados sobre história pregressa, uso de medicações e alergias. O próximo item (Avaliação) menciona os dispositivos de oxigênio, alimentação, edemas, eliminações, sondas, drenos, drogas infundidas e sinais vitais. E, por último, estão as (Recomendações), com espaço para registrar informações sobre as intercorrências do plantão, solicitações de avaliações pendentes, exames agendados e/ou cirurgias programadas com data e tipo de procedimento, a fim de facilitar os preparos necessários.

No item Avaliação, também é possível incluir imagem do curativo, através da opção da câmera fotográfica, ou anexar imagens feitas anteriormente, para armazenamento em um banco de dados. Isso permite acompanhar a evolução do paciente, de acordo com a Figura 2.

**Figura 2:** Tela registro de enfermagem, tela para inserir as informações do paciente e tela galeria de curativos do aplicativo Passando Plantão, Maringá, Paraná, Brasil, 2022

Todos os procedimentos que serão realizados podem ser cadastrados na tela inicial, na qual as informações ficarão armazenadas, de modo que, no próximo registro, não será necessário cadastrá-las novamente. São informações importantes que contemplam, por exemplo, uso de uma nova medicação, drenos, diurese, entre outros.

Após o término do preenchimento das informações, o usuário poderá salvar o documento ou imprimi-lo, gerando um arquivo em formato PDF, conforme evidenciado na Figura 3.

**Figura 3:** Tela relatório de enfermagem em formato PDF no aplicativo Passando Plantão, Maringá, Paraná, Brasil, 2022

O conteúdo do protótipo de aplicativo foi validado por 11 especialistas, a maioria do sexo feminino (91,7%), casados (75%), com idades entre 30 e 39 anos (41,7%), 40 e 49 anos (41,7%), oriundos do Paraná (83,3%). O tempo de formação profissional variou de 10 a 43 anos, com média de 18,2 anos de formados. Eram doutores com experiências e prática clínica em passagem de plantão 75% dos especialistas e 41% possuíam trabalhos em hospitais públicos (33,3%) e outros (66,7%).

Eles avaliaram o App nos seguintes quesitos: complexidade relacionada ao uso, funcionalidade, integridade das funções e dificuldades, apontando sugestões de melhora da qualidade do conteúdo.

A média total do escore do SUS foi de 79 pontos, considerada uma classificação excelente de avaliação de usabilidade do produto^(17)^, conforme apresentado na Figura 4.

**Figura 4:** Índice de validação do protótipo passando plantão conforme avaliação dos especialistas através da Escala SUS, Maringá, Paraná, Brasil, 2022

A meta de classificação utilizada é um escore de 68 pontos na escala, podendo alternar os valores dessa pontuação de acordo com as respostas dos especialistas. Observou-se, entre os 11 especialistas, que nove obtiveram respostas acima da média: 1, 2, 3, 4, 5, 6, 7,10 e 11. Apenas dois (especialistas 8 e 9) pontuaram abaixo da média, com 37,5 e 42,5 respectivamente.

Quanto ao nível de concordância das respostas, utilizou-se o CVC, pois avalia quantos especialistas concordaram ou discordaram das respostas apresentadas^(20)^. A questão 4 obteve menor índice de CVC 0,6. Considerando a questão “Eu acho que necessitaria de uma assistência técnica para utilizar o aplicativo”, infere-se que possa ter ocorrido viés na interpretação pelos especialistas, pois houve muita diferença entre as respostas.

Após análises de todas as questões realizadas no *software Microsoft Office Excel*, o CVC total do questionário foi de 0,7, considerado pelos autores como aceitável para avaliação, devendo ficar entre 0,7 e 0,8^(20)^, conforme demonstrado no Quadro 1.

**Quadro 1:** Cálculos do Questionário SUS e Coeficiente de Validade de Conteúdo

PARTICIPANTES	QUESTIONÁRIOS									
	Q1	Q2	Q3	Q4	Q5	Q6	Q7	Q8	Q9	Q10
Especialista 1	4	4	4	4	3	4	4	3	4	4
Especialista 2	2	4	4	4	3	3	3	4	2	4
Especialista 3	4	3	3	3	3	3	3	4	3	4
Especialista 4	4	4	4	4	3	4	2	4	4	4
Especialista 5	4	3	3	1	4	3	4	3	4	3
Especialista 6	4	3	4	4	4	4	4	4	4	3
Especialista 7	4	4	3	4	4	4	4	4	3	4
Especialista 8	2	1	1	3	1	1	2	1	1	3
Especialista 9	3	3	3	3	3	3	3	3	3	4
Especialista 10	4	3	4	0	4	4	4	4	4	4
Especialista 11	4	4	4	4	4	4	4	4	4	3
MÉDIA	3,5	3,3	3,4	3,1	3,3	3,4	3,4	3,5	3,3	3,6
CVCi	0,7	0,7	0,7	0,6	0,7	0,7	0,7	0,7	0,7	0,7
Pei	0	0	0	0	0	0	0	0	0	0
CVCf	0,7	0,7	0,7	0,6	0,7	0,7	0,7	0,7	0,7	0,7
Cálculo CVCt do Questionário SUS = 0,8 (Média do CVCi - Média do Pei)										

*Legenda: CVCi: Coeficiente de Validade de Conteúdo Inicial, Pei: Probabilidade de Erro Inicial, CVCf: Coeficiente de Validade de Conteúdo Final, CVCt: Coeficiente de Validade de Conteúdo Total.*

## DISCUSSÃO

O protótipo de aplicativo desenvolvido teve como proposta facilitar a passagem de plantão de enfermagem, através de informações claras e organizadas sistematizadas pelo método SBAR. O intuito foi tornar a comunicação eficaz, garantindo um processo de comunicação efetiva e, consequentemente, diminuição de EAs.

Em relação ao uso da metodologia SBAR para passagem de plantão, destacam-se dois estudos^(20)^. O primeiro, realizado para direcionar e agilizar a passagem de plantão entre os enfermeiros gestores de uma unidade de terapia intensiva pediátrica, possibilitou o direcionamento e agilidade na comunicação entre os profissionais de enfermagem, priorizando as informações mais importantes. Além disso, permitiu qualificar e padronizar os itens de segurança verificados^.^ O segundo, desenvolvido em duas unidades de terapia intensiva na Noruega em 2016, sobre o uso da metodologia SBAR na comunicação multiprofissional, demostrou que ambas as equipes notaram melhoras significativas na comunicação, segurança e assertividade na assistência ao paciente^.^

Com o aumento do uso da tecnologia da Informação e Comunicação (TiCs) por meio do uso dos *smartphones, tablets* e computadores, em diversas áreas, inclusive da saúde, essa ferramenta tornou-se uma grande aliada na tomada de decisão clínica e na qualidade da prestação de cuidados de enfermagem, entre outros^(22)^.

Nesse sentido, o uso da tecnologia aliado à gestão dos serviços melhora a comunicação entre a equipe, otimizando tempo e organizando as informações em tempo integral. O aumento de tecnologias voltadas para o profissional de saúde é evidente nos últimos anos: passou de 82% em 2019 para 88% em 2021. Tal crescimento mostra que, cada vez mais, os profissionais buscam na tecnologia uma forma de garantir confiabilidade, facilitar o fluxo de dados e informações, estabelecer rotinas e protocolos, além de qualificar o processo de assistir^(09)^.

O protótipo de aplicativo constitui uma inovação tecnológica em saúde, por tornar-se um App fundamentado nas necessidades do usuário final (público-alvo). O método centrado no usuário estabelece a participação/colaboração entre os usuários e dos *designers*/pesquisadores na fase de concepção para o desenvolvimento de sistemas informatizados, reconhecendo-os como partícipes deste processo de construção, o que impactará na adesão e uso futuro da aplicação em seus cotidianos de trabalho^(22,23)^.

Os enfermeiros estão receptivos à aquisição de novas tecnologias móveis que auxiliem o trabalho otimizando tempo e diminuindo falhas de comunicação. As tecnologias móveis são facilitadoras da sistematização e acesso a informações sobre pacientes e o processo de cuidar^(24)^. Nesse sentido, o protótipo fica evidenciado e tem esse objetivo, tendo os especialistas deixado clara a sua importância durante a passagem de plantão.

*A princípio, acredito que ele atende bem ao que foi proposto, para a passagem do plantão está ótimo.* (Especialista 7)

*Aplicativo autoexplicativo, possui todos as informações necessárias para a passagem de plantão da equipe de enfermagem.* (Especialista 12)

No Brasil, diversos pesquisadores vêm trabalhando no desenvolvimento de tecnologias que qualifiquem o cuidado e promovam segurança ao paciente. Pode-se mencionar a elaboração de um *software* com a finalidade de aplicar, de forma fácil e segura, o processo de enfermagem a pacientes assistidos em uma unidade de terapia intensiva, usando como base a Teoria das Necessidades Humanas Básicas, da enfermeira Wanda de Aguiar Horta, adotando os diagnósticos de enfermagem da NANDA-Internacional^(23)^.

Ainda em relação ao uso de tecnologias inovadoras na área de assistência com o objetivo desenvolver um *software* para a segurança do paciente em pediatria, estudo concluiu que a aplicação dessas ferramentas favorece a qualificação da assistência de enfermagem ao paciente pediátrico, oferecendo-lhe mais segurança durante a internação^(25)^.

Outros aplicativos existentes na literatura demostram eficiência e boa adesão ao uso dessas tecnologias. Cita-se o *WebEnf* utilizado para assistência de enfermagem, o qual possui 120 Procedimentos Operacionais Padrão (POPs) que abordam os cuidados e procedimentos de enfermagem. Tal aplicativo foi implantado no serviço de saúde público de uma grande emergência do Rio de Janeiro e demonstrou viabilidade e agilidade ao encontrar protocolos para o preparo do material antes de iniciar o procedimento de enfermagem^(26)^.

Pesquisa avaliou a aceitação de um aplicativo móvel para prevenção de lesão por pressão, identificando precocemente os pacientes em risco para o surgimento através da aplicação da escala de Waterlow e direcionando as medidas preventivas de acordo com o escore. O uso da ferramenta proporcionou a otimização do tempo dos profissionais e planejamento para prática de ações em prol da prevenção efetiva e individualizada para cada paciente, ao promover uma assistência segura e de qualidade. Os questionários de aceitação revelaram alta usabilidade do aplicativo pelos profissionais^(27)^.

O enfermeiro, na condição de gestor, tem a função de avaliar de forma contínua os serviços de enfermagem, em busca de alternativas para melhorar a qualidade da assistência. Sabe-se que o uso da tecnologia aliado à gestão dos serviços melhora a comunicação entre a equipe, otimizando tempo e organizando às informações em tempo integral^(27)^.

Dessa forma, identificar tecnologias capazes de melhorar a comunicação entre os profissionais nas instituições de saúde tem sido essencial. Essas tecnologias só têm a contribuir com os processos de trabalho, já que são apontadas como facilitadoras no processo de comunicação e disseminação de informações.

### Limitações do estudo

Destaca-se a dificuldade de operacionalização por ser tratar de um protótipo, podendo seu uso ser dificultado em setores onde o sinal de internet não seja de qualidade, principalmente para carregamento de imagens. Outro ponto a ser considerado foi o fato de não ter sido avaliada a usabilidade (na prática) do instrumento, por falta de tempo hábil. Porém, essa análise deverá ser realizada em estudo futuro para examinar a otimização do tempo, organização das informações e avaliação da equipe de enfermagem.

### Contribuições para a área da Enfermagem

Este estudo vislumbra benefícios para profissionais inseridos no mercado de trabalho, no sentido de facilitar o processo de comunicação entre os enfermeiros, uma vez que estabelece linguagem sistematizada durante a passagem de plantão. A pesquisa desenvolvida traz ainda reflexão ainda sobre a importância da usabilidade deste recurso digital em âmbito hospitalar, como ferramenta capaz de impulsionar a comunicação eficaz e a tomada de decisão.

## CONCLUSÕES

A construção do protótipo de aplicativo móvel para a passagem de plantão de enfermagem e sua validação contribuem para uma melhor padronização e estruturação das informações com ênfase no paciente, podendo melhorar a qualidade e segurança destas informações. A adoção de novas tecnologias pode auxiliar na comunicação entre a equipe, favorecendo a continuidade da assistência do paciente. No entanto, são necessários estudos futuros para implementação do protótipo de aplicativo móvel na prática dos enfermeiros, com o intuito de avaliar a eficácia das informações repassadas, otimização do tempo e, consequentemente, a redução dos EAs causados por falhas durante a comunicação.
